# Infant progressive colonic stenosis caused by antibiotic-related *Clostridium difficile* colitis – a case report and literature review

**DOI:** 10.1186/s12887-018-1302-9

**Published:** 2018-10-09

**Authors:** Xiaolong Xie, Bo Xiang, Yang Wu, Yiyang Zhao, Qi Wang, Xiaoping Jiang

**Affiliations:** Department of Pediatric Surgery, West China Hospital of Sichuan University, Chengdu, Sichuan Province China

**Keywords:** Colonic stenosis, *Clostridium difficile*, Colonitis

## Abstract

**Background:**

Colonic stenosis is a rare cause of pediatric intestinal obstruction. The root cause underlying colonic stenosis is unclear and there is no fixed operation.

**Case presentation:**

We reported on a male infant with progressive colonic stenosis caused by antibiotic-related colitis. The infant was admitted to our hospital with pneumonia but developed progressive abdominal distension and diarrhea following antibiotic treatment with meropenem. Initial testing of stool culture showed a *Clostridium difficile* infection. Additional testing with barium enema imaging showed stenosis at the junction of the sigmoid and descending colon at first and another stenosis occurred at the right half of the transverse colon 3 weeks later. Staged surgical treatment was performed with primary resections of the two parts suffering stenosis, ileostomy, and secondary intestinal anastomosis. A pathological exam then confirmed the diagnosis of colonic stenosis and the patient had an uneventful recovery and has been recovering well as evidenced by the 1-year follow-up.

**Conclusions:**

Based on a review of the literature and our case report, we found that progressive colonic stenosis caused by colitis due to antibiotic-related *Clostridium difficile* infection is rare in infants. Infants with colitis and repeated abdominal distention, vomiting, and constipation should be treated with the utmost caution and screened. Despite this, clinical manifestations depended on the severity of the stenosis. Barium enema, colonoscopy, laprascopy or laparotomy and colonic biopsy are helpful for diagnosis and differential diagnosis. While both one-stage and multiple-stage operations are feasible, a staged operation should be used for multiple colonic stenoses.

## Background

Colonic stenosis is a rare cause of pediatric intestinal obstruction. The root cause underlying colonic stenosis is unclear. Both congenital and acquired colonic stenoses (e.g post-necrotizing enterocolitis) has been reported on [1–29]. Here we present an infant case of progressive colonic stenosis after antibiotic-related Clostridium difficile colitis and a review of the related literature, which to the best of our knowledge had not been reported previously.

## Case presentation

**Fig. 1:**
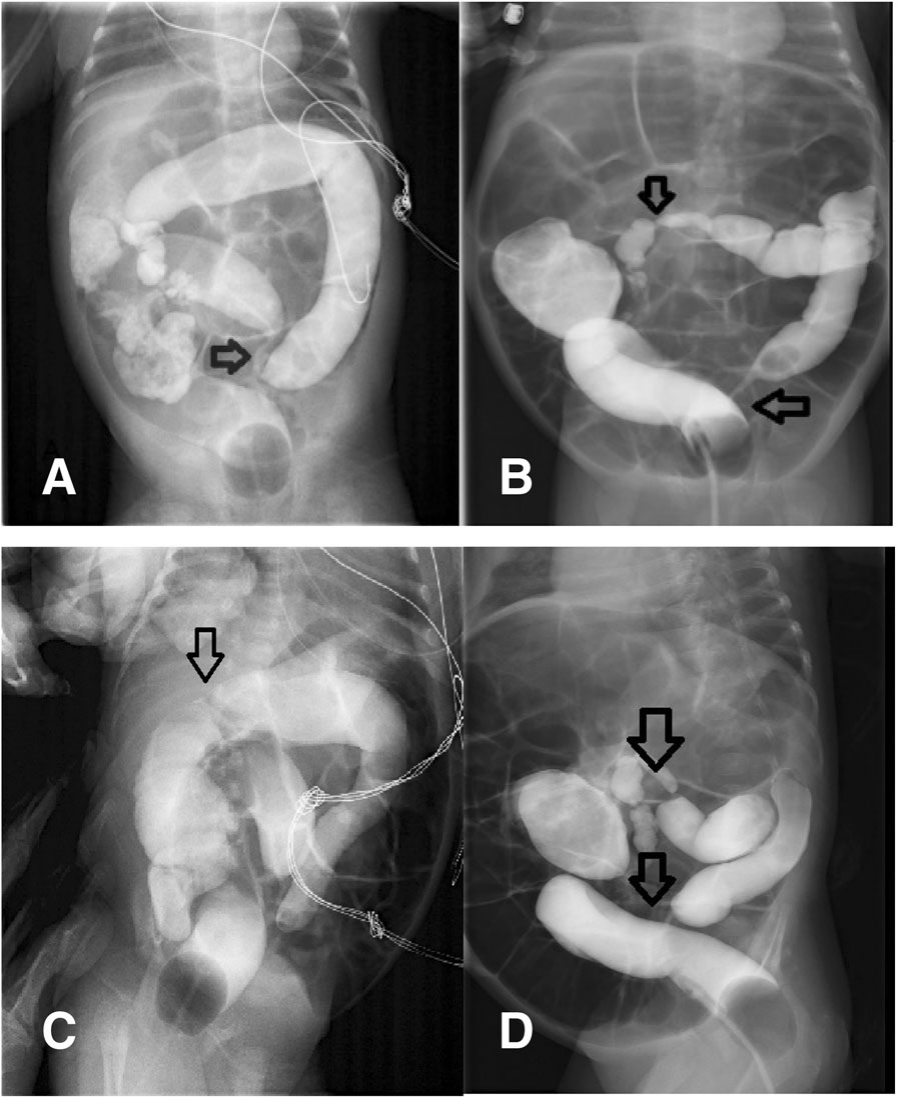
**a** Stenosis at the junction of the sigmoid and descending colon at the first barium enema. **c** No stenosis at the hepatic flexure at the first barium enema. **b** and **d** Stenoses at the junction of the sigmoid and descending colon and right transverse colon at the second barium enema

This male infant was the second child of a 39-year-old mother and was born via cesarean section during the 38th week of the pregnancy with a birth weight of 3300 g. The infant was admitted to our hospital 10 days after birth due to pneumonia and was treated with meropenem. He developed abdominal distension and diarrhea gradually from the 10th day of therapy on and stool culture revealed a *Clostridium difficile* infection. This was considered to be antibiotic-related and oral metronidazole and vancomycin were given. His symptoms were soon resolved but after discharge he gradually developed abdominal distension and constipation. A barium enema exam on the 42nd day after birth showed stenosis at the junction of the sigmoid and descending colon and a distended proximal bowel (Fig. 1a). Abdominal distension and constipation became more severe after 3 weeks of conservative treatment. A second barium enema exam then revealed another stenosis of the right transverse colon in addition to the previous stenosis (Fig. 1b).

**Fig. 2:**
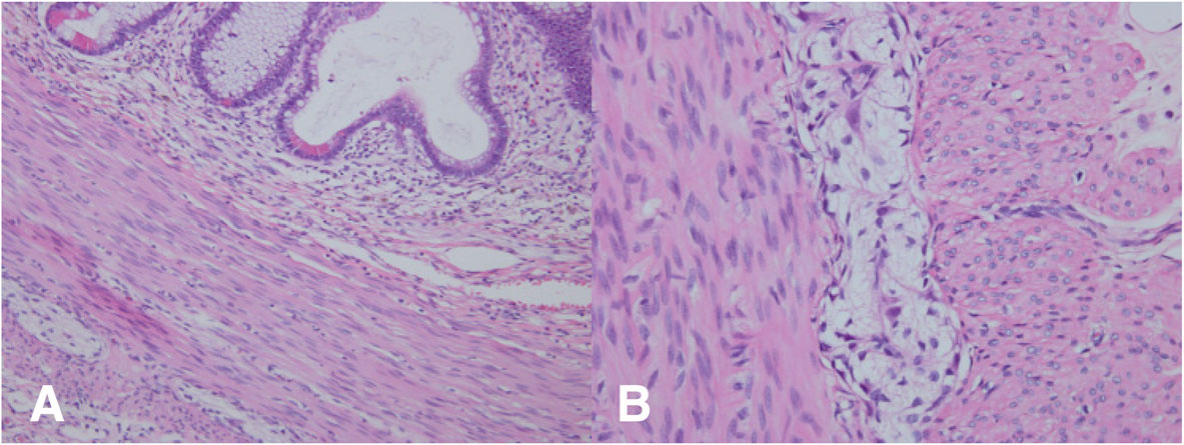
**a** Fibrosis of lamina propria of the narrow segment. **b** Normal ganglion cells

Primary surgical exploration revealed two segments of stenoses. One was at the junction of the sigmoid and descending colon and was 3.5 cm in length, while the other one was at the right transverse colon and was 4 cm in length. The small intestine, however, was still intact. Both the two parts were resected and an ileostomy was conducted at the terminal ileum. A pathological exam showed fibrosis of lamina propria in the narrow segments. Ganglion cells were normal (Fig. 2a and b). Closure of ileostomy was performed 3 months later and he made uneventful recovery. At the 1-year follow-up, he exhibited a normal dietary intake and defecation. His state of growth and development was in the 70th percentile.

## Discussion and conclusions

Intestinal obstruction caused by colonic stenosis is rare in children. We reviewed literature relating to pediatric colonic stenoses since 1961 (Tables 1 and 2) [1–29]. Pathogenesis of pediatric colonic stenoses varied among patients but congenital stenosis featured prominently among the literature. George Ekema and Reyes C introduced cases with congenital cytomegavirus (CMV) infection which involved the gastrointestinal tract and finally developed into colonic stenosis [30, 31]. Many researchers accepted the theory that fetal intestinal injury in the uterus due to disturbance in the blood supply was key. The causes of ischemia included emboli originating in the placenta, fetal herniation, kinks, intussusceptions, drugs (particularly cocaine) and placental causes [7, 10, 14, 16, 32–34]. Some colonic stenoses were secondary to necrotizing enterocolitis (NEC) [17–29], which were the most common type of non-congenital colon stenoses. The present case did not have complications during the pregnancy and perinatal period though. TORCH exam of the child and the mother was also negative. Colonic stenoses developed after antibiotic-related cololitis caused by *Clostridium difficile* progressively, which was confirmed by two barium exams. Sahara K and Kawaratani H reported that it is adult inflammatory bowel disease that causes colonic stenosis, and stool culture suggests *Clostridium difficile* [35, 36], but in our case the underlying disease wasn’t present. This patient did have a history of intestinal infection of Clostridium difficile prior to onset of symptoms though. The second barium exam showed a new site of stenosis compared to the first barium exam. This evidence shows that stenoses occurred secondary to infection rather than being congenital.

**Table 1:** Literature review of infant congenital colonic stenosis from 1961 to 2016

Authors	Year	Age	Localization	Surgical approach	Length of Stenosis
Zambaiti et al. [1]	2016	2 m	Ascending and transverse	II	Not described
Saha et al. [2]	2013	1.5Y	Descending	I	Not described
Galván-Montaño et al. [3]	2010	3Y	Ascending	I	5.0 cm
Ruggeri et al. [4]	2009	4 m	Ascending	–	Not described
Mizuno et al. [5]	2003	Newborn	Descending	I	Not described
García Vázquez et al. [6]	2002	2 m	Sigmoid	I	Not described
Abu-Judeh et al. [7]	2001	–	Ascending	I	4.0 cm
Dalla Vecchia et al. [8]	1998	Newborn	Not described	–	Not described
		Newborn	Not described	–	Not described
Murphree et al. [9]	1992	Newborn	Sigmoid	I	Not described
Sax [10]	1991	–	Descending-sigmoid	II	Not described
Pai GK and Pai PK [11]	1990	4 m	Rectosigmoid junction	I	Not described
Rescorla and Grosfeld [12]	1985	–	Not described	II	Not described
		Newborn	Sigmoid	II	9.5 cm
Schiller et al. [13]	1979	Newborn	Descending	II	3.0 cm
		Newborn	Sigmoid	II	4.0 cm
Erskine [14]	1970	2 d	Descending-sigmoid	II	16.0 cm
Benson et al. [15]	1968	–	Sigmoid	I	Not described
SANTULLI and BLANC [16]	1961	–	Sigmoid	II	Not described

I: Resection of stenotic segment and primary anastomosis II: A staged approach

**Table 2:** Literature review of colonic stenosis secondary to necrotizing enterocolitis treated by surgery

Authors	Year	patient	Localization of colon	Surgical approach
Marseglia L et al. [17]	2015	1 case	sigmoid	I
		28 cases with 46 stenoses	5 ascending	I
Gaudin A et al. [18]	2013	including 32 colonic stenosis	7 transverse	I
			20 descending	I
			1 whole colon	II
Pelizzo G et al. [19]	2013	3 cases	1 transverse	II
			1 right	II
Martinez-Ferro M et al. [20]	2009	11 cases	–	I
			10 right colon	I
Baudon JJ et al. [21]	1997	15 cases with 26 stenoses	5 transverse	I
			11 left colon	I
Vilariño Mosquera A et al. [22]	1995	15 cases	–	I
Schimpl G et al. [23]	1994	21 cases	–	I
Gobet R et al. [24]	1994	22 cases	–	I
Radhakrishnan J et al. [25]	1991	9 cases	–	I
D’Agostino S et al. [26]	1988	1 case	1 sigmoid	I
Schwartz MZ et al. [27]	1982	7 cases	7 left colon	I
Kosloske AM et al. [28]	1980	6 cases	–	II
Bonte C et al. [29]	1977	3 cases	3 sigmoid	I

I: Resection of stenotic segment and primary anastomosis II: A staged approach

It has been suggested that infants who have had abdominal distension, vomiting, and constipation should be suspected to suffer from colon stenosis. The barium enema was important in the diagnosis colonic stenosis in this case as it could determine the site of obstruction and severity of stenosis. However, a colonoscopy would be an alternative method to help with diagnosis [6]. The major differential diagnosis was Hirschsprung’s disease confirmed by pathological exam. In humans and other mammals, both domestic and wild, *Clostridium difficile* takes hold of the large intestine. While toxigenic and nontoxigenic strains do exist, toxigenic forms are responsible for causing disease in humans. Toxin A (TcdA) and toxin B (TcdB), are two closely related diarrhea-causing toxins and their presence is seen is a cause of pathogenicity. TcdB is found in all toxigenic strains, regardless of whether TcdA occurs concurrently. In addition to this, inactivation of Rho GTPases through enzymatic glucosylation of a conserved threonine residue is a similar molecular mechanism of action found in both of these toxins. Most often actin depolymerization and cell death follow, and the mechanism leads to the stimulation of an inflammatory cascade, with the end result being tissue damage, diarrhea, and pseudomembranous colitis [37, 38]. Moreover, significantly correlated with this tissue damage, diarrhea, and pseudomembranous colitis was the occurrence of a progression to fibrosis at the lamina propria.

Surgery is the major treatment of colon stenosis (Tables 1 and 2). For stenoses in both the right and left half of the colon, resection and primary anastomosis or proximal diversion could be successfully performed [1–29]. Pelizzo G reported three cases of colonic stenoses with norovirus infection in preterm babies. All patients received primary ileostomy followed by an immediate or staged coloectomy. Proximal diversion of intestinal contents is recommended to help to preserve colon integrity [19]. In our patient, primary resections of strictures of the colon with proximal diversion had successfully preserved the rest of the colon. This was important as the colonic stenoses was proved to be progressive in this case. The main reason why we chose to perform ileostomy rather than colon anastomosis was due to the fact of the colon stenosis being progressive. We didn’t know whether new stenosis would occur. Barium enema imaging before enterostomy didn’t reveal another colonic stenosis and the patient had an uneventful recovery followed by a clean check of health at the 1-year follow-up.

Based on a review of the literature and our case report, we found that progressive colonic stenosis caused by colitis due to antibiotic-related *Clostridium difficile* infection is rare in infants. Infants with colitis and repeated abdominal distention, vomiting, and constipation should be suspected and screened. Clinical manifestations depended on the severity of the stenosis. Barium enema, colonoscopy, laprascopy or laparotomy and colonic biopsy are helpful for diagnosis and differential diagnosis. Both one-stage surgery and multiple-stage operations are feasible, however staged operation should be used for multiple colonic stenoses.

## Abbreviations

CMV: Congenital cytomegavirus
NEC: Necrotizing enterocolitis
